# Managing Syncope After Transcatheter Aortic Valve Replacement: More than Meets the Eye

**DOI:** 10.19102/icrm.2020.110307

**Published:** 2020-03-15

**Keywords:** Bundle branch reentrant tachycardia, syncope, transcatheter aortic valve replacement

In this issue of *The Journal of Innovations in Cardiac Rhythm Management*, Kocherla et al.^1^ describe an interesting and educational case of bundle branch reentrant tachycardia (BBRT) in a patient with transcatheter aortic valve replacement (TAVR). Historically, BBRT has been described in patients with dilated cardiomyopathy: Caceres et al.^2^ in 1989 reported a 6% incidence of BBRT among patients with ventricular tachycardia (VT) over an eight-year period. Of note, 85% of their patients with BBRT had a history of syncope and all patients had underlying conduction disease with prolonged H–V intervals. Subsequent reports have suggested that BBRT can occur in patients with valvular heart disease, especially after corrective surgery in the setting of underlying conduction disturbances.^3^ TAVR has become an established therapeutic option not only in patients with severe aortic stenosis at high risk for adverse surgical outcomes but also in patients at low risk.^4^ Conduction abnormalities including left bundle branch block (LBBB), right bundle branch block (RBBB), and complete heart block occur at significantly higher rates in patients undergoing TAVR relative to surgical valve replacement.^5^

Given the increasing volumes of TAVR and the high incidence of conduction abnormalities recorded post-TAVR, the case described by Kocherla et al. raises several important questions and concerns in this population as follows.

## Who is at high risk for bundle branch reentrant tachycardia?

Kocherla et al.’s case differs from the classic description of BBRT in that the patient’s left ventricular function was normal. However, the patient in this case did have syncope—presumably from the BBRT. So far, to our knowledge, only three cases of BBRT have been described post-TAVR.^1,6,7^ All three reported cases occurred in the setting of underlying conduction disease post-TAVR. Further, of these three cases, two had RBBB and left anterior hemi-block and one demonstrated alternating BBB, all had residual atrioventricular (AV) conduction at the time of BBRT, and two had permanent pacemaker implantation at the time of BBRT. Prior reports have observed BBRT in up to 30% of patients with inducible VT following valve surgery.^4^ Of note, 40% of patients in this study had normal left ventricular (LV) function. The incidence of RBBB in patients with BBRT after valve surgery was 40% as compared with 6% among patients with dilated cardiomyopathy and BBRT.^4^ Based on these observations, normal LV function or RBBB do not preclude the development of BBRT in patients after TAVR. When patients develop RBBB following TAVR, it is likely that there is additional injury to the left bundle branch that causes significant conduction delay between the fascicles, increasing the susceptibility of reentry in these patients.

## What is the true incidence of bundle branch reentrant tachycardia?

While it is difficult to assess the true incidence of BBRT post-TAVR, our suspicion is that this is likely to be higher than that observed following surgical aortic valve replacement. The incidence of conduction abnormalities post-TAVR is as high as 65%. While the incidence of sudden death was reported to be 1% to 1.8% at two years post-TAVR,^8^ ventricular arrhythmias occur at much high rates in patients with LBBB post-TAVR.^9^ Careful monitoring of patients and the evaluation of stored events in patients with pacemakers are likely to shed additional light in this regard.

## What is the potential time frame in which bundle branch reentrant tachycardia can occur?

In previous observations of VT after valve surgery, BBRT was most likely to occur within the first one to four weeks when compared with the very late occurrence of myocardial VT.^3^ Surgical trauma–induced conduction delays and hyperadrenergic states following surgery are potential substrate/triggers for BBRT. In three cases of BBRT following TAVR, VT occurred at three, five, and 14 days following the procedure.^1,6,7^ It is likely that a similar kind of vulnerability exists in patients undergoing TAVR.

## Can bundle branch reentrant tachycardia occur in patients with complete atrioventricular block following transcatheter aortic valve replacement?

None of the cases reported had complete heart block post-TAVR. However, BBRT had been reported in a patient with complete heart block eight years after surgical aortic valve replacement who was successfully mapped and ablated.^10^ In the majority of patients with BBB occurring after TAVR, conduction delay occurs primarily in the main His bundle. Even if complete interruption of conduction occurs in the proximal His bundle, the distal his bundle–left bundle–right bundle junction may effectively function as the turnaround site for reentry. Is conduction delay/block occurring at the main His bundle during TAVR or is surgical aortic valve replacement alone an adequate substrate to develop BBRT? Does the patient need additional conduction delay in the peripheral bundle branches to facilitate unidirectional conduction block and reentry? We recently reported that the site of block is at the intra-Hisian level in three-fourths of patients with infranodal AV block.^11^ It is possible that, if the conduction disease extends into the His bundle–bundle branch junction, then reentry is unlikely to occur. In our experience, permanent His-bundle pacing was feasible in 63% of 46 patients with AV block post-TAVR, suggesting that the distal His bundle is intact in these patients.^12^ If so, the true incidence of BBRT may be much higher than thought and under-recognized. Further research into the mechanisms of BBRT in patients with conduction disease post-TAVR is warranted.

## What is the ideal treatment for bundle branch reentrant tachycardia?

It has been well-established that BBRT can be successfully ablated by targeting the right bundle branch. In patients with normal LV function and BBRT, the overall survival rate is much higher than in those with underlying cardiomyopathy following the catheter ablation of BBRT. In patients with underlying cardiomyopathy and BBRT, often, myocardial VT coexists and implantable defibrillators (ICDs) are likely to provide additional protection. In the absence of underlying cardiomyopathy or inducible myocardial VT, ICD implantation may not be necessary.

## What type of pacing should be considered?

A significant number of patients undergoing TAVR have left ventricular hypertrophy, left ventricular diastolic and/or systolic dysfunction, and heart failure symptoms. Right ventricular pacing in such patients places them at an increased level of risk for the onset or worsening of heart failure. In a recent study of 1,629 patients undergoing TAVR, it was demonstrated that patients requiring permanent pacemakers showed a heightened risk for heart failure hospitalization and lesser LV ejection fraction improvement relative to patients without pacemakers.^13^ Importantly, this effect was more pronounced in patients with reduced LV ejection fraction pre-TAVR.^14^ It is likely that, in patients with reduced LV function requiring permanent pacemakers, cardiac resynchronization therapy utilizing biventricular pacing or conduction system pacing may improve clinical outcomes. Even in the absence of underlying LV dysfunction, conduction system pacing (His-bundle pacing or left bundle branch pacing) is more likely to preserve ventricular synchrony when compared with right ventricular pacing and to reduce future adverse clinical outcomes.^15^ In our experience, His bundle pacing is more successful in patients with SAPIEN valves (Edwards Lifesciences, Irvine, CA, USA) than CoreValve systems (Medtronic, Minneapolis, MN, USA), while left bundle branch pacing is feasible in most patients post-TAVR.^12^

## How should we approach syncope in patients after transcatheter aortic valve replacement?

The recent description of BBRT in patients post-TAVR highlights the importance of establishing an accurate diagnosis regarding the etiology of syncope. While the majority of patients who present with syncope have demonstrable conduction disease in the form of RBBB, LBBB, or intermittent AV block, few may present with normal QRS intervals. In patients who present with high-grade or complete AV block with correlating symptoms, AV block is the most likely etiology of syncope and electrophysiology study in this context may not be necessary. However, in patients without demonstrable AV block with correlating symptoms, electrophysiology study may be warranted. The presumption of AV block as the cause of syncope in patients with underlying LBBB or RBBB may not be accurate. Patients with normal QRS and slightly prolonged P–R intervals may still have His-Purkinje disease and are likely to have substrate for AV block or BBRT. The completion of a thorough and complete electrophysiology study including isoproterenol challenge may be warranted to exclude the possibility of BBRT in these patients.

While BBRT is not a very common occurrence, we may see the incidence increase in the face of expanded indications for TAVR, especially in the setting of RBBB or LBBB following TAVR. An electrophysiology study to accurately diagnose the etiology of syncope is warranted in patients post-TAVR. The treatment of BBRT should be the ablation of the right or left bundle branch. If this ablation results in heart block or if the patient has significant His–Purkinje disease, the pursuit of biventricular pacing or conduction system pacing, especially if the LV function is reduced, should be considered. If the ablation is successful, LV function is normal, and no sustained myocardial VT is induced, ICD implantation is generally not necessary.
