# Supplementary material for: Managing Syncope After Transcatheter Aortic Valve Replacement: More than Meets the Eye
Source: J Innov Card Rhythm Manag. 2020 Mar 15;11(3):4037–40. doi: 10.19102/icrm.2020.110307 (PMC7192139; doi:10.19102/icrm.2020.110307)
Supplement: Related texts

# Article commentary: Drs. Rao and Tung consider

**Authors:** Swati Rao, Roderick Tung

**Affiliations:** ^1^Department of Medicine, Section of Cardiology, Center for Arrhythmia Care, Pritzker School of Medicine, the University of Chicago, Chicago, IL, USA

TAVR is emerging as a leading treatment option for both high- and low-risk surgical candidates who suffer from severe aortic stenosis.^1–3^ While heart block is the common sequelae and concern after valve deployment, Kocherla et al.^4^ present a case in which the predominant arrhythmia was VT arising within the conduction system. After undergoing TAVR, the patient was observed to show alternating bundle branch with high-degree AV block and underwent dual-chamber pacemaker implantation. Three days after the placement of a pacemaker and discharge from the hospital, she returned with syncope and was found to have VT with device-confirmed ventriculoatrial dissociation suggestive of BBRT. The authors present an excellent discussion of classical criteria to confirm bundle branch reentry (BBR).

BBRT is a macro-reentrant circuit with anterograde activation typically progressing down the right bundle branch and retrograde conduction occurring through the left branch,^5^ which is typified by a LBBB pattern similar or identical to baseline QRS. BBRVT clinically presents as syncope or sudden cardiac death and commonly develops in patients with structural heart disease such as ischemic or nonischemic cardiomyopathy, valvular heart disease, or myotonic dystrophy.^6^ It is important to note that the majority of patients who undergo permanent device implantation after TAVR are prescribed pacemakers, which are not protective against VT. BBRVT requires a critical level of conduction disease and slowing within the normally rapid conduction system, allowing for unidirectional block and an excitable gap. The prolongation of proximal His–V shown in this case is the classical setup of requisite conduction delay needed for reentry. In all reentrant arrhythmias, a central theme of obligate slow conduction is ubiquitous (eg, slow–fast atrioventricular nodal reentry tachycardia, BBR to initiate atrioventricular reentrant tachycardia, scar-related VT with delayed and late potentials as a result of deceleration).

Considering the two approved valves (ie, SAPIEN from Edwards Lifesciences, Irvine, CA, USA and CoreValve or Evolut R from Medtronic, Minneapolis, MN, USA), the self-expanding mechanism of the latter has been reported in the Ambulatory Electrocardiographic Monitoring for the Detection of High-degree AV Block in Patients with New-onset Persistent LBBB After TAVR (MARE) trial^7^ to accompany greater numbers of patients with persisting LBBB both at one year and overall due to the ongoing radial force projected onto the aortic annulus. In general, however, reports of tachyarrhythmias were rare, only occurring in 14% of the study population. With both types of valves, impingement on the root in turn injures and creates necrosis in the His bundle and proximal branching bundle on the basal LV septum, resulting in conduction delay (ie, intermittent bundle branch and BBR). Calcification and stretch from the prosthetic valve in addition to prior conduction disease (long P–R interval) in the patient likely attributed to her developing both brady- and tachyarrhythmia.^8^

## Can this unusual sequela be predicted before transcatheter aortic valve replacement?

As expected, the presence of preexisting RBBB and long H–V interval are predictive of subsequent higher-degree infra-Hisian AV block.^9^ This patient was noted to have a narrow QRS complex with a P–R prolongation of 212, which is presumably a reflection of AV nodal disease with a long A–H interval. However, it is possible that preexisting H–V disease may have been present, though knowledge of this is unlikely to change the management decision with respect to the risk for BBRVT. One could question whether there was any validity to conducting an electrophysiological study at the time of pacemaker implantation to exclude VT and BBR, based on this report.

We agree with the authors’ decision not to upgrade to an ICD after successful ablation of the right bundle. As BBR is a unique form of VT that is highly amenable to catheter ablation, ICD implantation in the absence of established primary prevention criteria or inducible myocardial VT is not warranted.^10^ The establishment of RBBB after ablation in the setting of LBBB without complete heart block nicely highlights that the vast majority of patients do not have “true” complete conduction block within the left bundle but, rather, show intraventricular conduction delay or intact retrograde conduction without antegrade conduction. A recent study by Chen et al. reports six patients who underwent electroanatomic mapping prior to ablation to demonstrate the nonstructural etiology of BBRT.^11^ In the case described by Kocherla et al., the authors primarily focus on the etiology of the BBRVT from the TAVR valve; however, electroanatomic mapping could have provided additional insight on any structural abnormalities and enhanced the accuracy of termination.

In summary, the authors present a unique case that beautifully illustrates both a common and uncommon arrhythmia presentation after TAVR. It should serve to remind us that conduction slowing is the classical prerequisite for BBR and to consider tachycardia etiologies along with anticipated bradycardia in patients who suffer recurrent arrhythmic symptoms after TAVR.

## References

[1] Mack MJ, Leon MB, Thourani VH (2019). Transcatheter aortic-valve replacement with a balloon-expandable valve in low-risk patients. N Engl J Med..

[2] Leon MB, Smith CR, Michael J (2016). Transcatheter or surgical aortic-valve replacement in intermediate-risk patients. N Engl J Med..

[3] Leon MB, Smith CR, Mack M (2010). Transcatheter aortic-valve implantation for aortic stenosis in patients who cannot undergo surgery. New Engl J Med..

[4] Tchou P, Mehdirad AA (1995). Bundle branch reentry ventricular tachycardia. Pacing Clin Electrophysiol..

[5] Kocherla C, Ward C, Horwitz PA, Mazur A (2020). Syncope after transcatheter aortic valve replacement: two faces of abnormal intraventricular conduction. J Innov Cardiac Rhythm Manage..

[6] Balasundaram R, Rao HB, Kalavakolanu S, Narasimhan C (2008). Catheter ablation of bundle branch reentrant ventricular tachycardia. Heart Rhythm.

[7] Rodés-Cabau J, Urena M, Nombela-Franco L (2018). Arrhythmic burden as determined by ambulatory continuous cardiac monitoring in patients with new-onset persistent left bundle branch block following transcatheter aortic valvereplacement: the MARE study. JACC Cardiovasc Interv..

[8] Young Lee M, Chilakamarri Yeshwant S, Chava S, Lawrence Lustgarten D (2015). Mechanisms of heart block after transcatheter aortic valve replacement—cardiac anatomy, clinical predictors and mechanical factors that contribute to permanent pacemaker implantation. Arrhythmia Electrophysiol Rev..

[9] Caceres J, Jazayeri M, McKinnie J (1989). Sustained bundle branch reentry as a mechanism of clinical tachycardia. Circulation..

[10] Singh G, Lahiri MK, Khan A, Schuger CD (2017). Bundle branch reentrant ventricular tachycardia after transcatheter aortic valve replacement. HeartRhythm Case Rep..

[11] Chen H, Shi L, Yang B (2018). Electrophysiological characteristics of bundle branch reentry ventricular tachycardia in patients without structural heart disease. Circulation Arrhythmia Electrophysiol..
